# Astrovirus induced nonpurulent encephalomyelitis in sheep: First report from Türkiye by high‐throughput sequencing

**DOI:** 10.1002/vms3.1499

**Published:** 2024-06-16

**Authors:** Yesari Eroksuz, Mehmet Ozkan Timurkan, Farzane Shams, Torsten Seuberlich, Burak Karabulut, Canan Akdeniz Incili, Emel Kara, Hatice Eroksuz

**Affiliations:** ^1^ Department of Pathology Faculty of Veterinary Medicine Firat University Elazig Turkey; ^2^ Department of Virology Faculty of Veterinary Medicine Firat University Elazıig Turkey; ^3^ Division of Neurological Sciences Vetsuisse Faculty University of Bern Bern Switzerland

**Keywords:** astrovirus, encephalitis, high‐throughput sequencing, immunohistochemistry, non‐purulent encephalomyelitiss

## Abstract

**Background:**

This study presents the case of non‐purulent encephalomyelitis associated with astrovirus infection in a sheep from Eastern Anatolia, Türkiye.

**Methods:**

A necropsy was performed on a sheep showing nervous signs. Afterwards, brain tissue samples were taken and examined with histopathological, immunohistochemical and molecular techniques.

**Results:**

Neuropathologic changes included neuronal degeneration, diffuse gliosis, multifocal perivascular cuffing, neuronophagy and neuronal necrosis in the cerebrum, the cerebellum and the cervical spinal cord. Aerobic and anaerobic bacterial culture, selective culture for *Listeria monocytogenes*, and PCR analysis for rabies virus, tick‐borne encephalitis virus, Türkiye encephalitis virus, small ruminant lentiviruses and border disease virus were negative. However, the presence of astrovirus RNA in cerebral, cerebellar and spinal cord samples was demonstrated by a pan‐astrovirus RT‐PCR. Immunohistochemical examinations revealed astrovirus antigens within the neuronal cytoplasm. High‐throughput sequencing techniques identified the causative agent as a member of the genotype species *Mamastrovirus* 13 but representing a distinct genetic lineage with similarity to ovine astrovirus 1 in the open‐reading frames (ORF)1ab region and muskox astrovirus in the ORF2 region.

**Conclusion:**

This report provides evidence that astroviruses are potentially encephalitis‐causing pathogens in ovine populations in Türkiye, featuring an astrovirus strain distinct from those previously identified in sheep.

## Graphical abstract

The case of non‐purulent encephalomyelitis associated with astrovirus infection in a sheep presented. Histopathological examination reveals signs of encephalitis, and astrovirus was detected by PCR.

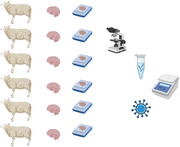

## 1 INTRODUCTION

Ovine farming in Türkiye holds a significant position in agriculture, given its economic impact and production value (Can, M.F. 2023). Yet, the threat posed by infectious diseases accentuates the importance of dedicated research to ensure animal health and stabilize economic gains. Notably, viral neuroinfectious diseases are particularly important in sheep husbandry (Yadav et al. 2019). Astroviruses belong to the Astroviridae family and are recognized for their positive‐sense single‐stranded RNA genome, the lack of an envelope and their relatively small size and star‐like appearance in electron microscopy (Mendez & Arias, 2013). Within the Astroviridae family, there are two genera: *Mamastrovirus*, comprising viruses that predominantly infect mammals, and *Avastrovirus*, comprising viruses that are found in avian species (De Benedictis et al., 2011). Astroviruses have been identified in more than 80 distinct host species, each specific astrovirus strain shows a preference for infecting either a single or a limited number of phylogenetically related species (De Benedictis et al., 2011; Wohlgemuth et al., 2019). They show substantial genetic diversity and are often associated with either asymptomatic infection or mild enteric disease. According to previous experimental research (Snodgrass et al., 1979), the virus replicates in the absorptive epithelial cells of the small intestine and may cause mild diarrhoea and partial villous atrophy in newborn gnotobiotic lambs. However, in mammals, astrovirus infections have also been associated with encephalitis, whereas in avian species, they have been linked to hepatitis and nephritis (De Benedictis et al., 2011; Mendez & Aries, 2013; Wohlgemuth et al., 2019). Although infrequent, sporadic cases of astrovirus associated encephalitis have been reported in humans (Bami et al., 2022) and various species, including, muskox (Boujon et al., 2019), cattle (Li et al., 2013; Schlottau et al., 2016), mink (Blomström et al., 2010), pigs (Arruda et al., 2017; Boros et al., 2017), sheep (Pfaff et al., 2017) and in alpacas (Küchler et al., 2020). In human populations, this condition is predominantly documented in immune‐compromised individuals and newborns (Quan et al., 2010).

Encephalitis linked to ovine astrovirus has been reported in three separate research studies, collectively involving three ewes and a lamb. (Boujon et al., 2017; Küchler et al., 2019; Pfaff et al., 2017). The first record of astrovirus‐induced encephalitis dates back to 2013 and concerned a 4‐year‐old Welsh Mountain ewe and a 10‐day‐old lamb from Wales (Pfaff et al., 2017). The second report was in a retrospective study in a 7‐year‐old Swiss white alpine ewe that died in 2004 (Boujon et al. 2017). The third report was on a case found retrospectively in an archived ovine case with non‐suppurative encephalitis from 1993 in Switzerland (Küchler et al., 2019). The viral genome sequences of all four cases are phylogenetically closely related and belong to the genotype species *Mamastrovirus* 13. Moreover, all four genomes were very similar to the genome sequences of strains found in brain tissues of two cows with neurological disease: bovine astrovirus (BoAstV) CH15 (Sueberlich et al., 2016) and BoAstV BH89/14 (Schlottau et al., 2016). This finding underlines the potential of these strains for cross‐species transmissions between sheep and cattle. In Türkiye, the enteric form of astrovirus infection had been identified previously in 4 out of 127 cases of bovine diarrhoea by RT‐PCR analysis (Turan & Isidan, 2018). However, up to the present time, there have been no recorded cases of astrovirus infection in sheep in Türkiye.

The aim of this study is to describe the molecular genomic characteristics and the related neuropathologic changes in an ovine case of nonpurulent encephalitis with astrovirus infection in Türkiye.

## 2 MATERIALS AND METHODS

### 2.1 Case history

The herd comprised around 400 Akkaraman sheep. Neurological signs were prominently observed in 20 lambs aged between 3 and 4 months and in 6 adult sheep aged between 2 and 3 years. Initial neurological signs appeared in the lambs during the colder seasons, and later, adult sheep began exhibiting similar signs. The primary clinical signs were lethargy, aimless walking, mastication of wool, circling actions and recumbency. The owner stated that there were no deaths related to these signs within the sheep population and that those animals displaying neurological signs showed a steady improvement over time. Three or four animals died, but it is unknown whether their deaths were related to this disease.

The owner also stated that the clinical signs of the disease lasted for about a week, and during this period, some of the affected animals remained recumbent for 4–5 days. They were hand‐fed with water and food and recovered completely from the clinical signs.

### 2.2 Necropsy

A 2‐year‐old female Akkaraman sheep, an indigenous sheep breed of Türkiye, was unable to stand. It was transferred to the pathology department by the owner for post‐mortem examination. After euthanasia and necropsy, representative tissue samples from the liver, the lymph nodes, the brain, spinal cord, lungs, the kidneys and the spleen were collected for histopathological, bacteriological and PCR examination.

### 2.3 Histopathology

Before being routinely processed, the representative tissue samples were fixed in a 10% solution of formalin, embedded in paraffin, sectioned at 4–5 μm, and stained with hematoxylin and eosin and Gram stains.

### 2.4 Immunohistochemistry

Immunohistochemistry using the anti‐open‐reading frames (ORF)2‐con polyclonal rabbit antiserum, originally designed to detect the capsid protein of BoAstV CH13, was conducted on tissue sections of the brain as described previously (Boujon et al., 2016). A brain tissue section from a BoAstV CH13 positive cow served as positive control and a brain tissue section of a healthy sheep as a negative control.

Additional staining for CD3, CD20 and CD68 was performed by using avidin–biotine complex method. Appropriate positive and negative controls were also prepared by using 3, 3′ diaminobenzidine as chromogen. Tissue sections were stained on an automated stainer (Ventana Medical System) for CD3 (mouse Dako‐M7254, 1:200 dilution) and mouse CD20 (L26, Cell Marque, 1:400 dilution) and anti‐mouse CD68 (Zeta Clone, KP1, Corporation, 1:200 dilution).

### 2.5 Bacteriology

Tissue samples from the brain, liver, spleen and kidney were freshly inoculated into aerobic cultures using blood agar (blood agar %7.0 sheep blood containing, CM0271, Oxoid), anaerobic cultures employing Cooked Meat Medium (CM0081, Oxoid) and a selective culture specifically for *Listeria monocytogenes* (CM0877, PALCAM Agar Base, Oxoid, UKand 75805, Oxford Agar, Sigma‐Aldrich).

### 2.6 Virus detection

PCR analysis was performed on the frozen samples from cerebrum and cerebellum to identify prevalent viruses responsible for encephalitis in sheep, including tick‐borne encephalitis virus, Türkiye sheep encephalitis virus, rabies virus, small ruminant lentiviruses and border disease virus. Subsequently, further examination was carried out, specifically targeting mammalian astroviruses. Cerebral and cerebellar specimens were subjected to viral nucleic acid extraction using the High Pure Viral Nucleic Acid Kit sourced from Roche, Germany. Subsequently, cDNA synthesis for viruses with RNA genomes was carried out employing the First Strand cDNA Synthesis Kit using random hexamers provided by Thermo Scientific. PCR analysis was performed using specific primers (Table 1) along with conditions referenced in relevant publications (Chu et al., 2008; Chen et al., 2018; Muz et al., 2013; Timurkan & Aydin, 2019).

**TABLE 1:** The primers applied in PCR and RT‐PCR assays the predicted amplicon sizes and associated references.

Virus	Primer (5′ − 3′)	Product size (bp)	References
**Tick borne encephalitis virus and Türkiye sheep encephalitis virus (pan*‐Flavivirus*)**	NS5/F‐GCMATHTGGTWCATGTGG	203	Alberdi et al. (2012)
	NS5/R‐GTRTCCCAKCCDGCNGTRTC		
**Small ruminant *Lentivirus***	LTR2s: TGACACAGCAAATGTAACCGCAAG	283	Muz et al. (2013)
	LTR2a: CCACGTTGGGCGCCAGCTGCGAGA		
**Border disease virus (pan‐*Pestivirus*)**	324‐ATGCCCWTAGTAGGACTAGCA	288	Timurkan & Aydın (2019)
	326‐TCAACTCCATGTGCCATGTAC		
**Pan‐Astrovirus**	ASTF1‐GARTTYGATTGGRCKCGKTAYGA	F1‐F2/R1: 424	Chu et al. (2008)
	ASTF2‐GARTTYGATTGGRCKAGGTAYGA		
	ASTR1‐GGYTTKACCCACATNCCRAA	NF1‐NF2/R1: 409	
	ASTNF1‐CGKTAYGATGGKACKATHCC		
	ASTNF2‐AGGTAYGATGGKACKATHCC		
**Rabies virus**	JW12 ATGTAACACC(C/T)CTACAATTG	JW12‐JW6mix: 606	Heaton et al. (1997)
	JW6 (DPL) CAATTCGCACACATTTTGTG		
	JW6 (E) CAGTTGGCACACATCTTGTG		
	JW6 (M) CAGTTAGCGCACATCTTATG	JW12‐JW10mix: 582	
	JW10 (DLE2) GTCATCAAAGTGTG(A/G)TGCTC		
	JW10(ME1) GTCATCAATGTGTG(A/G)TGTTC		
	JW10 (P) GTCATTAGAGTATGGTGTTC		

### 2.7 High‐throughput sequencing (HTS)

Formalin‐fixed paraffin‐embedded brain tissue (cerebellum and cerebral cortex) blocks were cut at 20‐μm thick sections. These sections were deparaffinized with xylol and total RNA was extracted using the RNeasy FFPE kit (Qiagen) according to the manufacturer's instructions.

The quantity and quality of the purified total RNA was assessed using a Thermo Fisher Scientific Qubit 4.0 fluorometer with the Qubit RNA BR Assay Kit (Thermo Fisher Scientific, Q10211) and an Advanced Analytical Fragment Analyzer System using a Fragment Analyzer RNA Kit (Agilent, DNF‐471), respectively. Thereafter, 1000 ng of input RNA was depleted of rRNA using a Human/Rat/Mouse HMR V2 kit (Lexogen, SKU 144) following the Lexogen User guide: 144UG288V0101. Next, cDNA libraries were generated using a CORALL RNA‐Seq V2 Library Prep Kit with UDI 12 nt Set A2 (Lexogen, SKU 172) according to the RTM protocol and 11 PCR cycles (Lexogen User Guide: 171UG394V0100). The resulting cDNA libraries were evaluated using a Thermo Fisher Scientific Qubit 4.0 fluorometer with the Qubit dsDNA HS Assay Kit (Thermo Fisher Scientific, Q32854) and an Agilent Fragment Analyzer (Agilent) with a HS NGS Fragment Kit (Agilent, DNF‐474), respectively. Pooled cDNA libraries were sequenced 100 bp single‐end using NovaSeq 6000 SP Reagent Kit v1.5 (100 cycles; illumina, 20028401) on an illumina NovaSeq 6000 instrument. The quality of the sequencing run was assessed using illumina Sequencing Analysis Viewer (illumina version 2.4.7) and all base call files were demultiplexed and converted into FASTQ files using illumina bcl2fastq conversion software v2.20. The quality control assessments, rRNA depletion, generation of libraries and sequencing were carried out at the Next Generation Sequencing Platform, University of Bern.

### 2.8 Bioinformatics

Raw reads were subjected to a quality control process using FastQC (Ver. 0.11.7) (Andrews, 2010). Reads were then quality selected and trimmed with fastp (Ver. 0.12.5) at the 5′ and 3′ ends with a Phred quality control score less than 25 to remove low quality reads and molecular identifiers, which were added during the library preparation. Trimmed reads were mapped to the sheep reference genome (ARS‐UI_Ramb_v2.0, GenBank) using STAR (Ver. 2.7.3a) (Dobin et al., 2012). Unmapped reads were retained and analysed by Genome detective (Ver. 2.12.2) (Tamura et al., 2021) (www.genomedetective.com) for the presence of viral sequences. Virus sequences were further analysed by Geneious prime function (Ver. 2023, Dotmatics).

### 2.9 Phylogenetic analysis

Phylogenetic analyses were done with the MEGA 11 software (Tamura et al., 2021). The nucleotide sequences as well as protein sequences of the of representative astroviruses of the genotype species *Mamastrovirus* 13 were acquired from Genbank and compared to those of the new astrovirus presented in this study. Sequences were aligned with ClustalW and the alignments were then further used to predict the best‐suited phylogenetic model using the model finder tool in MEGA 11: GTR+G+I for the nonstrucural‐proteins 1ab (nsp1ab) and the genome sequences and JTT + G for the capsid protein sequences. Phylogenetic trees were constructed using the maximum‐likelihood method and the above‐mentioned models with 1000 bootstraps replicates.

## 3 RESULTS

### 3.1 Gross and histological changes

**FIGURE 1:**
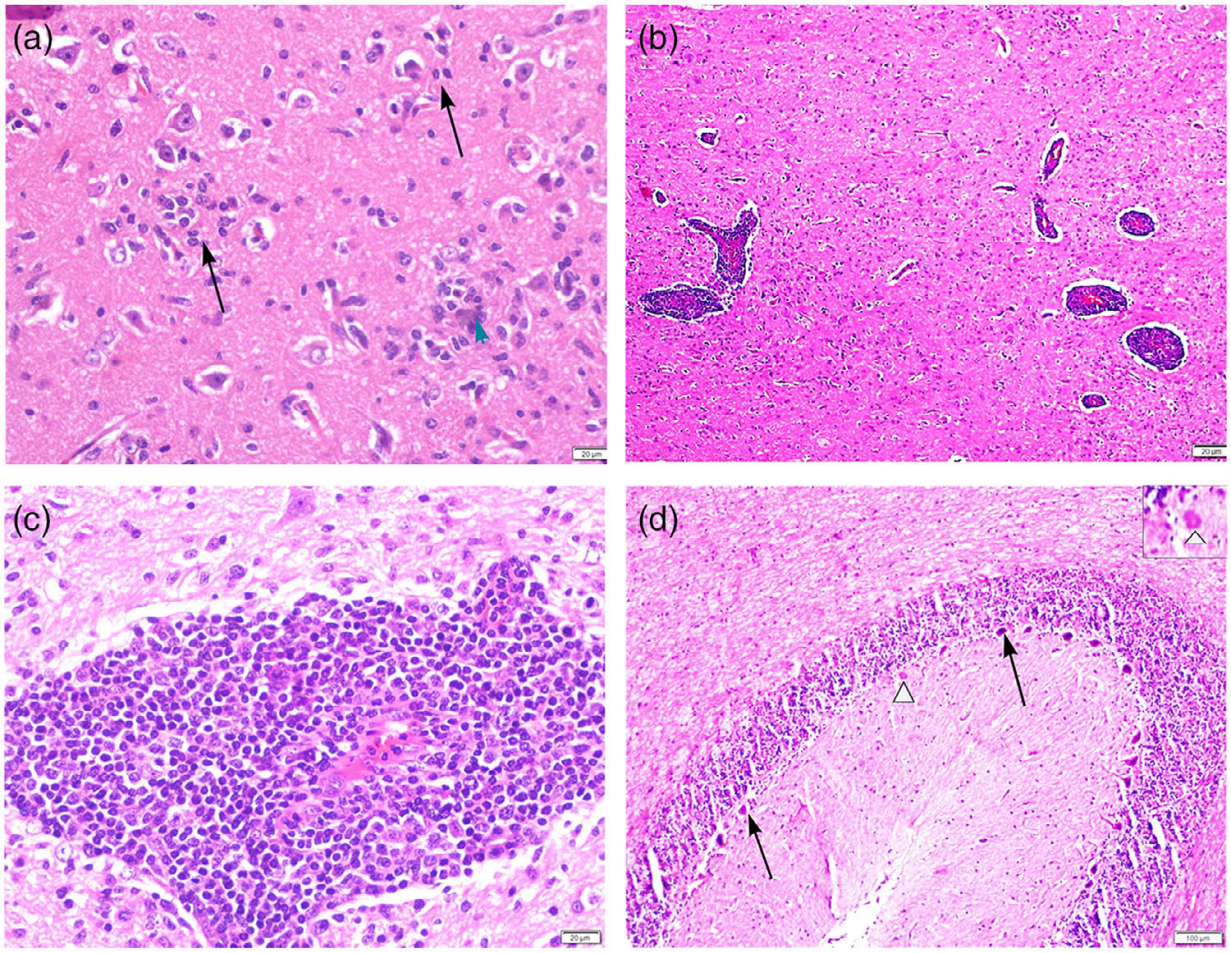
Histological findings. Formalin‐fixed tissue samples were sectioned at 4–5 μm and stained with hematoxylin and eosin (H&E). (a) In the pons, glial nodules (arrows) and neuronophagy (arrowhead) are observed. (b) Perivascular cuffs are multifocally distributed within the white matter of the medulla oblongata. (c) The medulla of the frontal lobe shows dense lymphohistiocytic cell infiltration, forming perivascular cuffs. (d) In the cerebellum, there is a reduced number of Purkinje cells between the indicated arrows, with a necrotic Purkinje cell highlighted by the arrowhead and detailed in the inset.

Gross examination revealed multifocal hydatid cysts in the dorsal diaphragmatic surface of the liver, with diameters ranging between 1 and 4 cm. Concurrently, the sheep was markedly emaciated. The histological findings were a non‐suppurative encephalomyelitis characterized by neuronal degeneration, gliosis and the presence of neuronophagic nodules (Figure 1A), perivascular lymphohistiocytic infiltrates (Figure 1B,C) marked gliosis, neuronophagia and satellitosis. Perivascular infiltrates consisted of up to 30 layers of cells in the Virchow–Robin spaces. Vascular endothelial cells were swollen and reactive. In the cerebellum, necrosis was evident in Purkinje cells, accompanied by their depletion (Figure 1D). The lesions were diffuse and distributed in both cerebral white and grey matter. Sections from different regions of the brain displayed these lesions. However, the brainstem showed the most severe lesions. Additionally, a mild to minimal lymphohistiocytic meningitis was present in the brain. The spinal cord showed the lesions within the grey matter, including focal gliosis and perivascular cuffing. No variation in lesion severity was observed across different segments of the spinal cord. The other lesions beyond the nervous system consisted of focal interstitial nephritis and diffuse moderate vacuolar degeneration in the liver.

### 3.2 Virological and bacterial investigations

In our study, the pan‐astrovirus primers (Alberdi et al., 2012) specifically target a partial region of the RNA‐dependent RNA polymerase (RdRp) gene within ORF1ab. Upon conducting partial sequence analysis of the amplicon, it was determined that the nucleotide similarity was 88.21% with the OvAstV1 strain (NC002469) and 89.95% with an *Ovibos moschatus* astrovirus strain (MOxAstV CH18, MK211323). The results of bacterial growth indicate the absence of cross‐contamination with bacteria.

### 3.3 Immunohistochemistry

**FIGURE 2:**
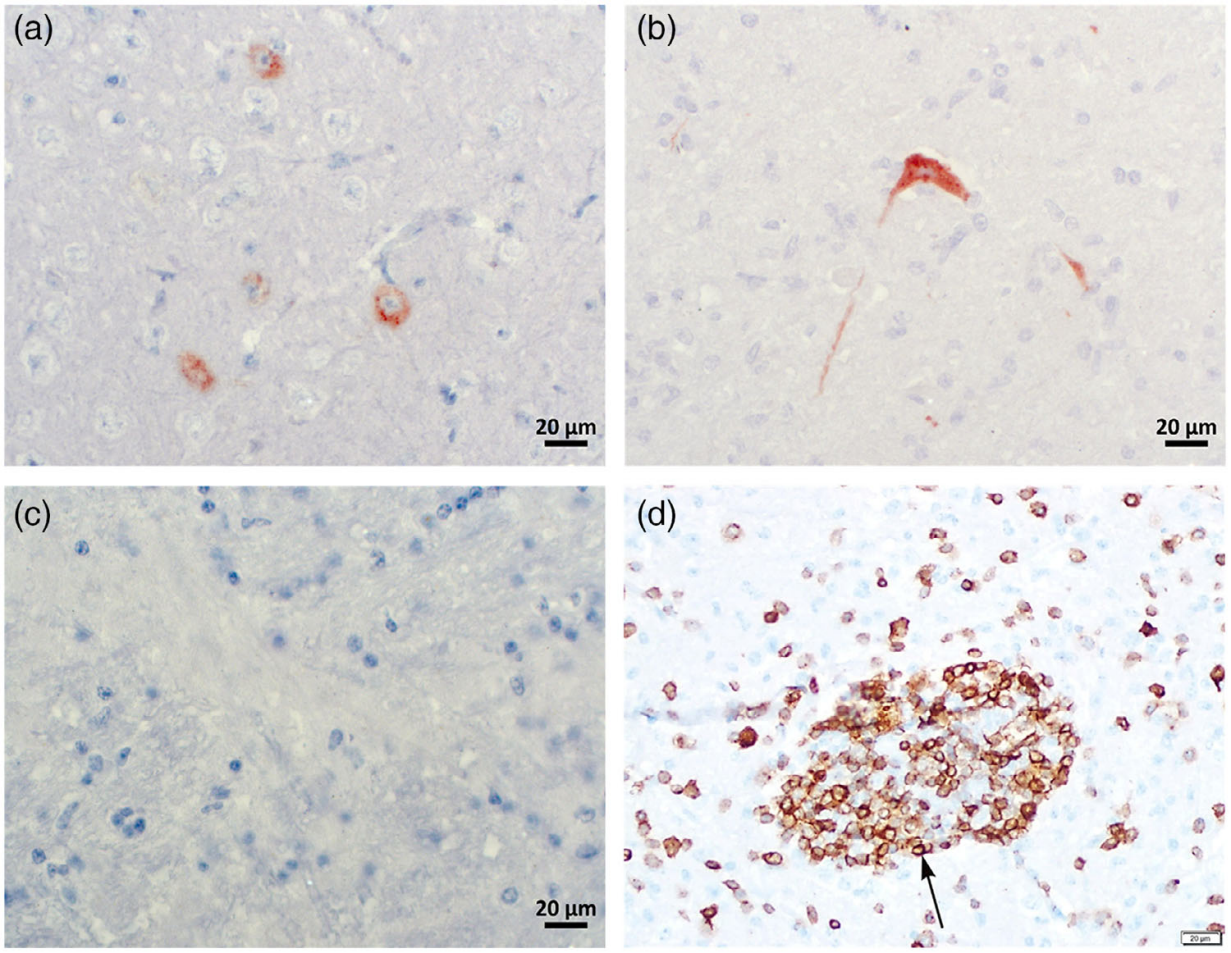
Immunohistochemistry findings. This analysis was conducted on tissue sections from both the cerebellum and the cerebral cortex. (a) Positive immunoreaction in neuronal cytoplasm in the cerebellum using the avidin–biotine complex (ABC) immunoperoxidase method. (b) Positive immunoreaction was observed in the neuronal cytoplasm of the cattle's cerebrum (positive control) using the ABC immunoperoxidase method. (c) Negative immunoreactivity in neuronal cytoplasm of the cerebrum in cattle (negative control) using the ABC immunoperoxidase method. (d) CD3‐positive immunoreactive cells in perivascular infiltrate in the cerebral section.

The presence of astrovirus capsid protein was demonstrated in neurons (Figure 2A) through immunohistochemistry in paraffine tissue section of the cerebellum. This was achieved using a rabbit polyclonal antibody generated against a peptide from the conserved region of the BoAstV CH13 capsid protein together with appropriate positive (Figure 2B) and negative controls (Figure 2C).

Immunohistochemically, the majority of the cells in perivascular infiltrates were CD3 positive (Figure 2D), with fewer being CD68 positive, and the least or absent were CD20 positive cells.

### 3.4 High‐throughput sequencing

To generate comprehensive genetic data on the astrovirus strain detected in the brain tissues using RT‐PCR and IHC, total RNA extracts from brain tissue were submitted to high‐throughput sequencing (HTS) and analysed through a web‐based bioinformatics virus discovery pipeline (Genome Detective). These analyses resulted in one viral contig of 6401 nt that was assembled from a total of 159,187 reads with a depth of coverage of ∼2000. This contig revealed a nucleotide identity of 87% to ovine astrovirus 1 (NC‐002469.1) with a genome coverage of 99%. It showed the typical astrovirus genome organization, featuring three (ORF1a, ORF1b and ORF2) along with a ribosomal‐slippery sequence [AAAAAAC] between ORF1a and ORF1b. The viral sequence is coding‐complete, whereas the termini of the genome were not further determined. No other viral sequences were detected by the virus discovery pipeline.

### 3.5 Phylogenetic analysis

The astrovirus strain identified in this study was designated ovine astrovirus TR‐FIRAT (GenBank accession 544230). A Blastx search revealed that the nsp1ab (encoded by ORF1ab) was found to be most similar (98.7% identity) to the nsp1ab of OvAstV‐1, whereas the sequence of the capsid protein (encoded by ORF2) displayed the highest similarity (96% identity) to that of Muskox astrovirus (MOxAstV) CH18 (Accession MK211323.1), but not to that OvAstV‐1 (75% identity).

**FIGURE 3:**
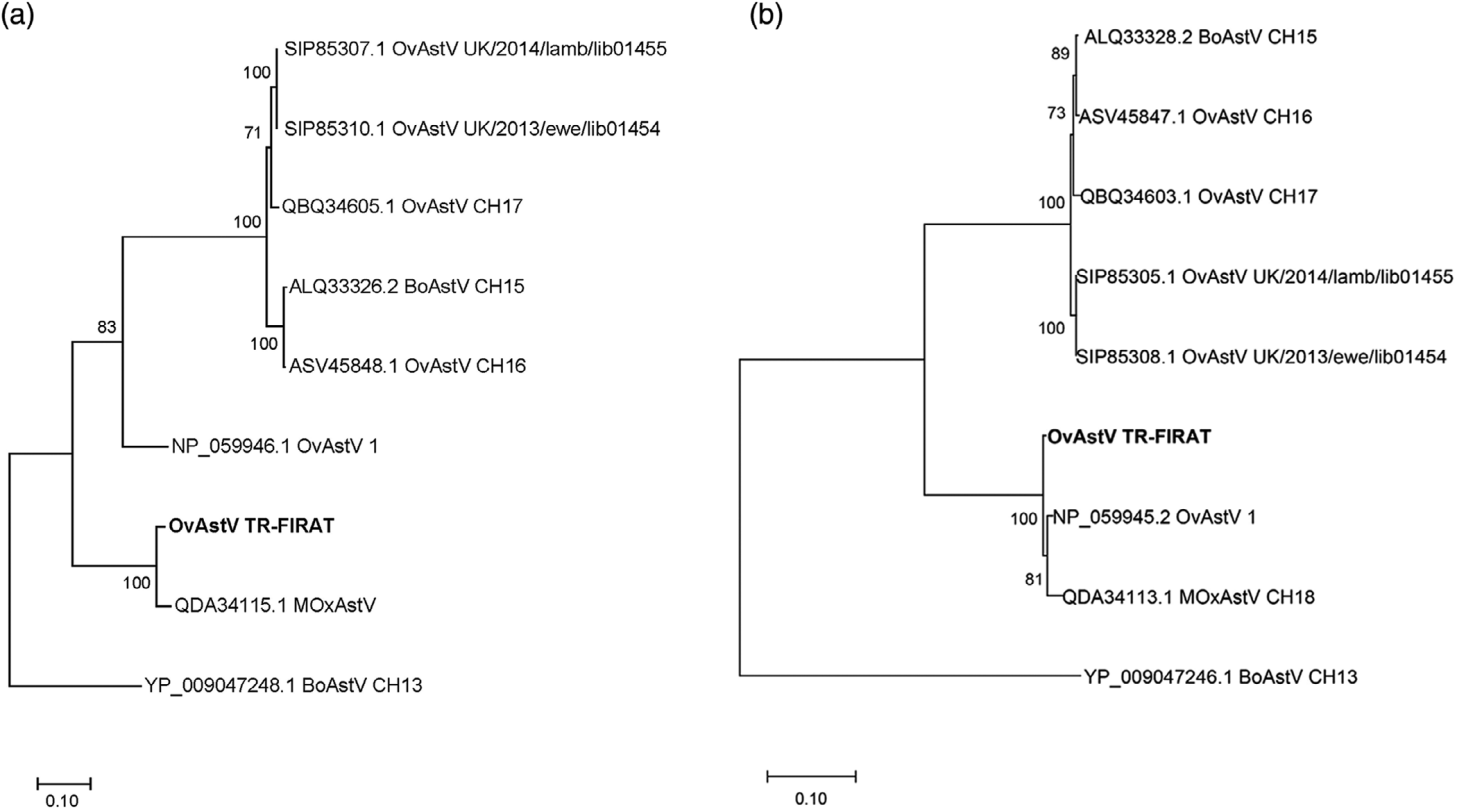
Phylogenetic comparison of nonstructural protein 1ab (nsp1ab) and capsid protein. (a) Phylogenetic comparison of the nsp1ab of the ovine astrovirus TR‐FIRAT strain identified in the present study with representative members of the genotype species *Mamastrovirus* 13. Genbank accession numbers are indicated. (b) Phylogenetic comparison of the capsid protein of the ovine astrovirus TR‐FIRAT strain, identified in the present study with representative members of the genotype species *Mamastrovirus* 13. Genbank accession numbers are indicated. BoAstV, bovine astrovirus; MOxAstV, muskox astrovirus; OvAstV, ovine astrovirus.

This relationship was further supported by phylogenetic comparison of the nsp1ab protein and the capsid protein with representative members of the genotype species *Mamastrovirus* 13 (Figure 3A,B).

## 4 DISCUSSION

In the last decade, there has been a considerable rise in reports on astroviral infections affecting extraintestinal tissues in mammals (Bami et al., 2022; Blomström et al., 2010; Boujon et al., 2017; Boros et al., 2017; Küchler et al., 2020; Pfaff et al., 2017; Quan et al., 2010; Schlottau et al., 2016). The potential of astroviruses to invade the central nervous system highlighted the concerns about the broader impact of these infections. However, the precise mechanisms underlying brain invasion are not understood. The present cases in a flock exhibited a brief duration of illness, like the acute presentations often seen with RNA viruses. These viruses typically replicate vigorously during the infection's early phases, leading to severe manifestations of disease. However, they may eventually be neutralized and eliminated by the host's immune defences. It is noteworthy that in specific human RNA viruses, including Ebola virus, measles virus and tick‐born encephalitis virus, viral RNA can remain within the host for extended periods, often taking refuge in areas referred to as immunologically privileged sites, less accessible to the immune system, such as the brain, heart muscle and eyes (Griffin, 2022). Neuroinvasive astroviruses might employ mechanisms to evade the immune response and to sustain their presence within the host. These mechanisms still need to be further investigated for astrovirus infections. RNA viruses ensure their longevity by infecting new hosts and establishing a prolonged presence in parts of the body less accessible to the immune system, like the brain, heart muscle and eyes. The continuous presence of viral RNA in these areas is crucial for their ability to infect new hosts and ensure their propagation (Griffin, 2022). Astroviruses, much like human RNA viruses, may also benefit from their ability to reach the brain, as it can provide protection against immune clearance, support long‐term persistence, and enable new transmissions strategies.

The histopathological findings in the presented case are the classical lesions of viral encephalomyelitis. As previously observed in sheep (Pfaff et al., 2017), cattle (Schlottau et al., 2016), mink (Blomström et al., 2010) and pigs (Boros et al., 2017), the lesions consisted of neuronal degeneration, gliosis and perivascular cuffings populated by T lymphocytes and macrophages. The significant influx of T lymphocytes is a distinguishing characteristic of typical viral encephalitis. This condition triggers T lymphocytes, especially CD8^+^ T cells, to move towards the central nervous system. These T lymphocytes can cross the CNS barrier and locating infected cells, aiding in clearing the virus (Ai & Klein, 2020). For differential diagnostic considerations, other etiological agents of viral encephalomyelitis, *L. monocytogenes* and *Coenurus cerebralis*, were evaluated. The histopathological diagnosis was confirmed through molecular studies and immunohistochemical staining in this study, polyclonal sera originally generated against the capsid protein of BoAstV CH13 also detected OvAstV TR‐FIRAT‐infected cells in brain sections. This suggests that despite marked genetic diversity among species, the antigenic structure of the capsid protein remains preserved in some aspects. Similarly, an earlier study has indicated that antisera against the capsid protein of BoAstV CH13 cross‐reacted with the capsid protein of muskox astrovirus CH18 (Boujon et al., 2019).

Although astroviruses are primarily recognized as pathogens of the enteric system, recent studies have underscored their neurotropic potential. In our research, the identified strain has been determined to be responsible for encephalitis in sheep. A previous research (Boujon et al., 2019) addressed both the crossing of the species barrier (from sheep to cattle and vice versa) and the identification of neurotropic astrovirus strains. The OvAstV TR‐FIRAT strain we detected, upon full genome analysis, exhibited a significant similarity (97.8%) in the ORF1 region to a known sheep astrovirus (OvAstV, accession no: Y15937). Meanwhile, the ORF2 region closely resembled (96%) that of a muskox the Muskox (MoxAstV, Accession no: MK211323). This observation indicates a probable recombination mutation, frequently encountered in astroviruses. However, due to the limited number of global studies on this strain and the relative genomic similarity, it cannot definitively be characterized as a recombination. A previous study (Boujon et al., 2019) explored the virus's capability to exhibit tropism across diverse tissues, ranging from the digestive system to the nervous system. They also observed that the virus operates independently of species‐specific movement. In their research, they identified a strain closely related to our detected strain in the ORF2 region, which was the Muskox strain. Similarly, in our region (Elazığ, Türkiye), although the strain detected in a sheep did not demonstrate immediate widespread prevalence. Future investigations can explore whether the astrovirus crosses species barriers and its level of affinity to the nervous system in conjunction with the enteric system.

HTS data has provided profound molecular insights into the astrovirus strain. This strain demonstrates a nucleotide identity of 87% to ovine astrovirus 1% and boasts 99% genome coverage, reflecting the typical astroviral genome organization. The presence of a coding‐complete viral sequence reinforces the validity of our findings, notwithstanding the undetermined genome termini. Phylogenetic analyses confirm that the detected astrovirus strain, named ovine astrovirus TR‐FIRAT, shares a close genetic relationship with established astrovirus strains. The notable similarity between the nsp1ab protein and OvAstV‐1, as well as the capsid protein and Muskox astrovirus, suggests the possibility of a recombination event or a shared evolutionary lineage.

The significant genetic diversity observed in ORF2 among OvAstV strains, clustered in separate clades, indicates that the structural capsid protein, encoded by ORF2, may promote the infection of a broad host range and is not associated with species‐specific identification. Conversely, the RdRp, identified in the polypeptide cleaved from the nsp1ab polyprotein (ORF1ab), has been conserved across species.

As a limitation of our study, it should be noted that while 26 out of 400 sheep have exhibited signs of encephalitis associated with astrovirus infection, the exact source of infection remains unknown. It is also unclear whether transmission occurred from a single source and affected those in close contact or if the virus spread through another unidentified source. Astroviruses have been previously detected in the faecal matter of wild forest rodents (Griffin, 2022) and bats (Yin et al., 2022), hinting at their potential roles as astrovirus reservoirs or hosts. Given the genetic similarity observed between the present virus and the one identified in muskoxen, transmission from the muskox might be a consideration. However, this species is not native to Türkiye. Interestingly, similar to the present report, in the earlier reports, the sheep were raised in high‐altitude region (Boujon et al., 2017; Quan et al., 2010; Pfaff et al., 2017).

Considering the two cases reports from Switzerland and another two in Wales, this report presents the fifth documented case of this disease in sheep. Importantly, the anamnesis findings from this study suggest that the postmortem diagnostic challenges in field conditions may stem from factors such as the full or partial recovery of affected animals, coupled with low or nonexistent mortality rates. This might also explain why there have been only a limited number of cases reported to date. Due to the circling behaviour of the animals, owners might suspect ‘circling disease’ related to *C. cerebralis* or *L. monocytogenes*, and the possibility of them not opting for a postmortem examination, can hinder the diagnosis of the disease.

## 5 CONCLUSION

Although rare, encephalitis caused by astroviruses is of significant concern due to its potential impact on animal husbandry, possible zoonotic transmission, and its ability for cross‐species transfer. This study highlights the need to identify astroviruses as differential diagnosis of encephalitis in ovine populations in Türkiye. However, the astrovirus reported in this study significantly differs genetically from those previously reported strains. Further studies are necessary to elucidate their epidemiology and clinical implications, enabling the development of targeted strategies for control and prevention.

## AUTHOR CONTRIBUTIONS

Yesari Eroksuz, Burak Karabulut, Canan Akdeniz Incili and Emel Kara performed necropsy and histopathological analyses. Mehmet Ozkan Timurkan, Farzene Shams and Torsten Seuberlich performed microbiologically and virological procedure and immunohistochemistry. Yesari Eroksuz, Torsten Sueberlich, Mehmet Ozkan Timurkan, Farzene Shams and Hatice Eroksuz conducted the analysis of the data and the writing of the article.

## CONFLICT OF INTEREST STATEMENT

The authors declared no conflicts of interest.

## FUNDING INFORMATION

There are no funders to report for this submission.

## CONSENT FOR PUBLICATION

We give our consent for the publication of identifiable details, which can include photograph(s) and/or videos and/or case history and/or details within the text (“Material”) to be published in the above Journal and Article.

### ETHICS STATEMENT

Not applicable.

### PEER REVIEW

The peer review history for this article is available at https://www.webofscience.com/api/gateway/wos/peer‐review/10.1002/vms3.1499.

## Data Availability

The authors confirm that the data supporting the findings of this study are available within the article [and/or] its supplementary materials.
